# Exposure to Acetylcholinesterase Inhibitors Alters the Physiology and Motor Function of Honeybees

**DOI:** 10.3389/fphys.2013.00013

**Published:** 2013-02-05

**Authors:** Sally M. Williamson, Christopher Moffat, Martha A. E. Gomersall, Nastja Saranzewa, Christopher N. Connolly, Geraldine A. Wright

**Affiliations:** ^1^Centre for Behaviour and Evolution, Institute of Neuroscience, Newcastle UniversityNewcastle upon Tyne, UK; ^2^Division of Neuroscience, Medical Research Institute, Ninewells Medical School, University of DundeeDundee, UK

**Keywords:** honeybee, honey bee, acetylcholinesterase inhibitor, acetylcholine, pesticide, motor function, coumaphos, aldicarb

## Abstract

Cholinergic signaling is fundamental to neuromuscular function in most organisms. Sub-lethal doses of neurotoxic pesticides that target cholinergic signaling can alter the behavior of insects in subtle ways; their influence on non-target organisms may not be readily apparent in simple mortality studies. Beneficial arthropods such as honeybees perform sophisticated behavioral sequences during foraging that, if influenced by pesticides, could impair foraging success and reduce colony health. Here, we investigate the behavioral effects on honeybees of exposure to a selection of pesticides that target cholinergic signaling by inhibiting acetylcholinesterase (AChE). To examine how continued exposure to AChE inhibitors affected motor function, we fed adult foraging worker honeybees sub-lethal concentrations of these compounds in sucrose solution for 24 h. Using an assay for locomotion in bees, we scored walking, stopped, grooming, and upside down behavior continuously for 15 min. At a 10 nM concentration, all the AChE inhibitors caused similar effects on behavior, notably increased grooming activity and changes in the frequency of bouts of behavior such as head grooming. Coumaphos caused dose-dependent effects on locomotion as well as grooming behavior, and a 1 μM concentration of coumaphos induced symptoms of malaise such as abdomen grooming and defecation. Biochemical assays confirmed that the four compounds we assayed (coumaphos, aldicarb, chlorpyrifos, and donepezil) or their metabolites acted as AChE inhibitors in bees. Furthermore, we show that transcript expression levels of two honeybee AChE inhibitors were selectively upregulated in the brain and in gut tissues in response to AChE inhibitor exposure. The results of our study imply that the effects of pesticides that rely on this mode of action have subtle yet profound effects on physiological effects on behavior that could lead to reduced survival.

## Introduction

Populations of honeybees and other pollinating insects have precipitously declined in many countries worldwide in the past 20 years. The spread of parasites and pathogens in domesticated honeybee populations has reduced the survival of managed colonies and eliminated wild colonies from some countries altogether. Exposure to new forms of pesticide that target insect acetylcholine receptors (AChRs) is also emerging as a major contributing factor in bee population decline (Le Conte et al., 2010; Maxim and van der Sluijs, 2010).

The decline in bee populations may represent an inability on the part of bees to adapt to multiple forms of stress from parasites, pathogens, and pesticides. In particular, major changes in the nature of the bee’s within-hive chemical environment have occurred as a result of the use of chemicals meant to target the parasitic mite, *Varroa destructor* (Rosenkranz et al., 2010). Acaricides accumulate inside the hive, with lipophilic compounds reaching very high levels in the comb wax as well as being present in the hive food stores (Mullin et al., 2010). The acaricide, coumaphos, is a lipophilic compound applied directly into hive boxes via strips (Milani and Iob, 1998; Rosenkranz et al., 2010); as a consequence, it is often the compound recorded at the highest concentrations in studies of in-hive pesticide residues in wax combs (Mullin et al., 2010; Wu et al., 2011). This acaricide has been widely used in commercial beekeeping operations in lieu of tau-fluvalinate, another acaracide to which most *Varroa* populations are now resistant (Milani, 1995; Rosenkranz et al., 2010). Coumaphos is also used to control infestations of another parasite of bee colonies, the hive beetle (Baxter et al., 1999).

Accumulated stress in response to disease, combined with exposure to environmental toxins, could precipitate the rapid decline of the honeybee (Hawthorne and Dively, 2011; Wu et al., 2012) but its role in the rapid disappearance of honeybees during the colony collapse disorder crisis remains uncertain (Johnson et al., 2009). Chronic exposure to acaracides or other pesticides could tip the balance in terms of whether a colony will adapt, or succumb, to other environmental stresses such as pathogens (Moser, 1995; Moye and Pritsos, 2010). Like coumaphos, many pesticides are lipophilic and accumulate in the comb wax in hives after bees come into contact with these substances on plants while foraging (Mullin et al., 2010; Wu et al., 2011). Two important classes of pesticide are the organophosphates (e.g., chlorpyrifos) and the carbamates (e.g., aldicarb) that, in common with coumaphos, are inhibitors of an important enzyme involved in neurotransmission, acetylcholinesterase (AChE), that hydrolyses acetylcholine (ACh) at the synaptic cleft (Pohanka, 2011). The inhibition of AChE leads to an excess of the Ach that results in prolonged activation of cholinergic receptors, followed by their desensitization (Fukuto, 1990; Pohanka, 2011).

The aim of this study was to investigate the physiological and behavioral adaptations of adult worker honeybees to prolonged, sub-lethal exposure to AChE inhibitors. Honeybees have two active forms of the AChE enzyme (Belzunces et al., 1988; Badiou et al., 2007); one form, AChE_m1_, is expressed at a much higher level in the bee’s head than the other (AChE_m2_; Belzunces et al., 1988). It is possible that chronic exposure to an AChE inhibitor could affect expression of these two enzymes. For this reason, we investigated whether exposure to AChE inhibitors caused alterations in AChE gene transcription in the honeybee brain and gut. To identify the influence of AChE inhibitors on motor function, we used an assay for locomotion (Maze et al., 2006) to quantify how AChE inhibitors affected locomotion, grooming, and the righting reflex.

## Materials and Methods

### Honeybees

Honeybee colonies (*Apis mellifera mellifera*) were obtained from stock of the National Bee Unit (FERA, York, UK). During the months of January to March 2012, bees were maintained in an indoor flight room at a temperature of 28°C, with a 12-h light/dark cycle. They were introduced to the flight room in early January, and the experiments executed from January to March 2012. During the months of May to July 2012, bees were kept outdoors and allowed to forage freely. The experimental procedure described below was carried out twice: the experiments were first performed on bees taken from the indoor winter colony, then repeated using bees taken from the outdoor summer colonies. Adult worker bees were collected in small plastic vials from outside the colony entrance. The vials were placed on ice until the bees were immobile, and the bees were transferred to small plastic boxes for exposure to pesticides as described below.

### AChE inhibitors

Coumaphos, chlorpyrifos, aldicarb, and donepezil were obtained as dry powders of >99% purity from Sigma-Aldrich. The compounds were dissolved in DMSO to produce stock solutions of 1 mM concentration. These stock solutions were then diluted to the working concentration to 10 nM in 1 M sucrose solution for the first set of experiments. A second experiment exploring the influence of concentration of coumaphos on behavior was performed using the following doses: 1 μM, 100 nM, and 10 nM. Preliminary experiments were performed to ensure that the drug concentrations chosen were sub-lethal and were readily consumed by bees. There was no difference in the survival rates (Kruskal–Wallis, $\chi_{6}^{2}$ = 6.894, *P* = 0.331) or food consumption (Kruskal–Wallis, $\chi_{6}^{2}$ = 4.13, *P* = 0.659) between treatment groups. Overall, mean consumption was 143 mg syrup per bee per 24 h. The LC_50_ for coumaphos was estimated in a previous study to be 751 ppm, though this method used topical application rather than oral dosing (Garrido et al., 2012). In our study, the coumaphos concentrations used equate to 363, 36.3, and 3.6 ppb; it has previously been reported that stored food in coumaphos treated hives has a mean coumaphos concentration of 180 ppb (Mullin et al., 2010), making the doses chosen here field relevant for oral consumption. Each honeybee from the different treatment groups therefore consumed approximately these drug doses: 0.56 ng of donepezil, 0.25 ng of aldicarb, 0.46 ng of chlorpyrifos, and 0.47, 4.7, or 47 ng of coumaphos.

### Exposure to AChE inhibitors

Acetylcholinesterase inhibitors such as coumaphos are administered directly in the colony and are soluble in wax and in food products within the colony (Mullin et al., 2010; Wu et al., 2011; Gomez-Perez et al., 2012). Feeding was chosen as the method of pesticide administration to reflect the concentrations encountered in stored food (Mullin et al., 2010); we have previously reported that coumaphos has effects on learning and memory after oral administration of low concentrations (Williamson et al., 2012). After capture from outside the colony, cohorts of 10–20 honeybees were placed in plastic boxes (16.5 cm × 11 cm × 6.5 cm) which had ventilation holes in the lid, and four holes in the sides to allow insertion of feeding tubes. The box lid had an additional hole of 2 cm diameter to allow capture of individuals for behavioral experiments; this remained covered with tape during the treatment period. Feeding tubes were made from 2 ml microfuge tubes with four ∼2 mm holes drilled along one side to allow the bees to insert their mouthparts into the feeding solution. The bees were allowed to feed *ad libitum* on treatment solutions in each box for 24 h. Control bees were fed 1 M sucrose; treatment groups were fed 1 M sucrose containing the appropriate concentration of AChE inhibitor (see information about concentrations above). Feeding tubes were weighed before and after the treatment regime to determine food consumption and therefore drug dose. Mortality at the end of the 24 h treatment was also recorded.

### Behavioral observations

Behavioral observations were recorded using a method modified from Maze et al. (2006). Individual bees were captured from each treatment box without anesthetization by placing a small plastic vial over the hole in the box lid and allowing the bee to crawl upwards into it. The captured bee was then transferred to a plastic Petri dish of 1.5 cm depth and 9 cm diameter, and allowed to acclimatize for 5–10 min. Behavior was recorded using the Noldus Observer software. Observation time was 15 min for each individual, and the frequency, mean duration, and percentage of the interval were recorded for each type of behavior. Behaviors which had been seen in pilot observations on coumaphos treated bees were recorded during the experiment. These behaviors were classified as walking, flying, remaining still, falling upside down, grooming the head, grooming the body, and unusual abdominal spasms and movements (which included rotating, dragging, and tucking the abdomen, and abdominal pulsations which preceded fecal expulsion).

### AChE assay

Honeybee brain and gut tissue were dissected and homogenized in PBS and protein concentrations determined by the Bradford assay; AChE activity was assayed at 14 μg/ml using the Ellman’s assay. AChE inhibitors (at appropriate concentration) were incubated for 30 min in the presence of color indicator 5′,5′-dithiobis-2-nitrobenzoic acid (286 μM), acetylthiocholine iodide substrate (0.86 mM); AChE activity was measured via spectrophotometer for absorbance at 412 nm. AChE activity was normalized to control measurements. IC50 values were obtained from Hill equation fits of the data from at least three independent replicates.

### PCR amplification of AChE transcripts

Semi-quantitative PCR was used to investigate whether AChE inhibitor treatment altered AChE gene transcript levels in the brain and gut of the honeybee. After AChE treatment (as described above) bees were cold anesthetized prior to dissection. Brain tissue and gut tissue were dissected, and the RNA extracted using Trizol^®^ (Invitrogen) reagent. Sample size for each extraction was brain tissue from five animals, or gut tissue from three animals, as these were the same tissue volume. Samples were collected three separate times for each treatment group. Extracted RNA was subject to treatment with DNase (Promega) to remove genomic contamination, then re-purified by phenol-chloroform extraction. Reverse transcription was carried out using Superscript II^®^ (Invitrogen), and random primers. PCR was performed using a Bio-Rad T100^™^ thermal cycler. The reaction conditions were as follows: an initial denaturation step at 95°C for 3 min, followed by 35 cycles of 95°C for 30 s, 55°C for 30 s, then 72°C for 30 s, with a final extension step of 72°C for 5 min.

PCR was performed using GoTaq^®^ Green Mastermix, and the following primers: β-tubulin control (accession no. XM_394471) forward primer, CAACGTGTACTACAACGAGG, reverse primer, 5′ TGGGTGACGGTACCACGGAG 3′; AChE-1 (accession number XM_393751), forward primer TATCTGCGAGTTCCCGTTCG, reverse primer GCTCCCTGCTCACCTTTA; AChE-2 (accession number NM_001040230), forward primer ACCCGAACACCAACATATCC, reverse primer CTGATCCCAAAGACCCATGT. To ensure that equal amounts of cDNA were present in each reaction, a mastermix was prepared containing 6 μl cDNA and sufficient reagents to perform 3 μl × 50 μl PCR reactions; this was mixed well and then 48 μl of the reaction mixture was transferred to three separate tubes for addition of the gene specific primers. PCR products were separated by agarose gel electrophoresis (2%), and visualized by GelRed^®^ (Biotium) staining. 12 μl of PCR product was loaded into each well. Gel images were obtained, and the relative brightness of DNA bands quantified, using the Bio-Rad Gel Doc^™^ EZ imager and Image Lab^™^ software (version 4.0). The relative quantification was performed within-sample: from each set of three reactions for each separate sample, β-tubulin was selected as the reference gene, and the relative brightness therefore assigned the value of 1. The brightness of the AChE-1 and AChE-2 bands from the same sample were then quantified relative to this.

### Statistical analysis

All analyses were performed using IBM SPSS software. To identify correlations in the behaviors for the time budgets (percentage of the interval spent performing behaviors) and to reduce the dimensionality of the data, we used a principal components method of factor analysis (Johnson and Wichern, 1992). The resulting factor scores generated for the factors with eigenvalues greater than 0.95 were then entered into a multivariate general linear model (MGLM). Multiple comparisons were calculated as least square differences (LSD). Bout frequency was analyzed using Poisson regression. Data for gene expression was analyzed using a non-parametric median test for independent samples with *post hoc* comparisons.

## Results

### AChE inhibitors disrupt motor function and cause abdominal spasms

A factor analysis was performed to identify correlations in the behaviors measured prior to further analyses: factor analysis produced four factors that accounted for 82.8% of the variation in the data (Table 1). The first factor, which accounted for 27.8% of the variation in behavior, was mainly representative of the inverse relationship between walking behavior, and upside down behavior and abdominal spasms. The second factor represented the time spent grooming, as it revealed a strong positive correlation between head grooming and body grooming. The third and fourth factors represented still and flying behaviors respectively and did not have strong correlations with other behaviors.

**Table 1:** **Factor analysis produced from whole behavioral dataset**.

	Factor			
	1	2	3	4
% Variance	27.8%	21.3%	19.2%	14.4%
Walk	−0.857	−0.163	−0.458	0.007
Head grooming	−0.008	0.817	0.277	−0.003
Body grooming	0.069	0.880	−0.132	−0.081
Still	0.157	0.064	0.936	−0.105
Fly	−0.101	−0.069	−0.093	0.987
Upside down	0.882	−0.108	−0.292	−0.113
Abdomen spasms	0.630	0.063	0.267	−0.058

*Shading indicates which behavioral variables made the largest contribution to each factor. Factors with eigenvalues greater than 0.95 were extracted from the data; all other factors explained 10% or less of the variation in the data, and so were not included. *N* = 120*.

**Figure 1:**
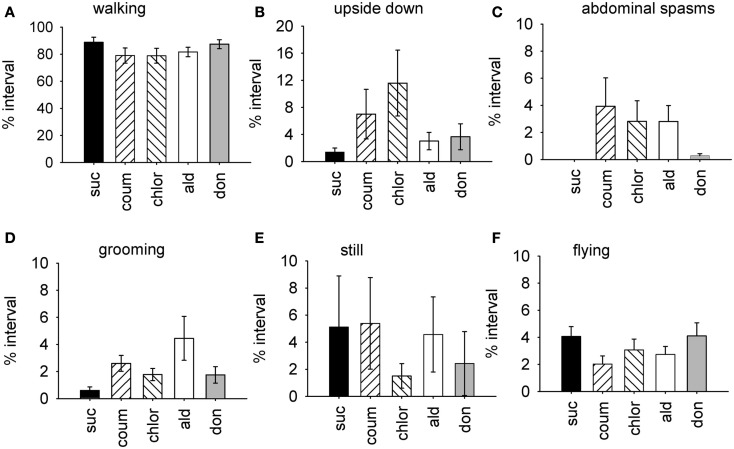
**Time budget data for all behaviors from bees treated with AChE inhibitors**. Graphs show the mean percentage of the observation interval (±SEM) spent engaged in different types of behavior. **(A)** Walking, **(B)** upside down, **(C)** abdominal spasms, **(D)** grooming (includes both head and body grooming), **(E)** still, and **(F)** flying. Data is pooled from both indoor winter and outdoor summer bee colonies. *N* ≥ 23 individual bees for each treatment group. Suc, sucrose; Coum, coumaphos; Chlor, chlorpyrifos; Ald, aldicarb; Don, donepezil.

**Figure 2:**
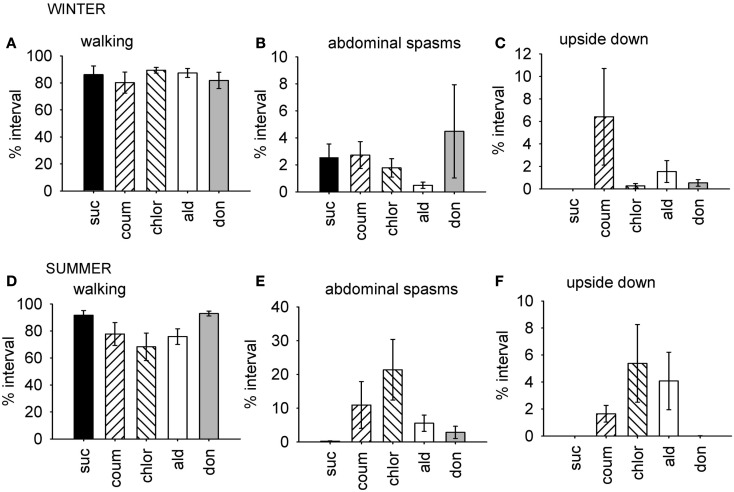
**Time budget data for behaviors which were differentially affected by AChE inhibitors according to the time of year**. Graphs show mean percentage of the observation interval (±SEM) spent engaged in the behaviors comprising factor 1, which were affected by AChE inhibitors in the summer, but not in the winter. **(A)** Walking, winter bees. **(B)** Upside down, winter bees. **(C)** Abdominal spasms, winter bees. **(D)** Walking, summer bees. **(E)** Upside down, summer bees. **(F)** Abdominal spasms, summer bees. *N* ≥ 11 for each treatment group at each time of year. Suc, sucrose; Coum, coumaphos; Chlor, chlorpyrifos; Ald, aldicarb; Don, donepezil.

The factor scores generated by the factor analysis were used to test how exposure to the AChE inhibitors affected the expression of behavior. The time of year that the bees had been used was also included in the analysis. As represented by factor 1, bees exposed AChE inhibitors spent slightly less time walking, and had difficulty righting themselves when they fell over (upside down; Figure 1). They also exhibited abdominal spasms, a behavior never observed in the control group. Furthermore, the expression of these behaviors depended on the time of year the bees were assayed (MGLM two-way interaction, *F*_4,119_ = 2.61, *P* = 0.039). These data were therefore split according to whether the bees had been taken from the indoor winter colony or the outdoor summer colony for further analyses (Figure 2).

For the winter bees, the behaviors represented by the first factor were not affected by AChE treatment (GLM, *F*_4,59_ = 0.951, *P* = 0.442), but the bees collected in the summer were (GLM, *F*_4,59_ = 2.61, *P* = 0.045). In summer, the chlorpyrifos treated bees engaged in less walking behavior and spent more time upside down and performing abdominal spasms and movements, relative to the control bees (LSD, *P* = 0.007). A similar trend was observed in the coumaphos and aldicarb treatment groups, but this was not significant.

Factor 2 mainly represented grooming behavior, and it was strongly influenced by AChE inhibitor exposure (MGLM, *F*_4,119_ = 2.79, *P* = 0.030), but this effect did not vary according to the time of year at which the experiment was performed (MGLM, *F*_4,119_ = 0.882, *P* = 0.477). Factor 3, the behavior of remaining still, and factor 4, flying behavior, were both unaffected by AChE inhibitor treatment (MGLM, factor 3, *F*_4,119_ = 0.543, *P* = 0.704; factor 4, *F*_4,119_ = 0.973, *P* = 0.425).

### AChE inhibitors increase grooming behavior

**Figure 3:**
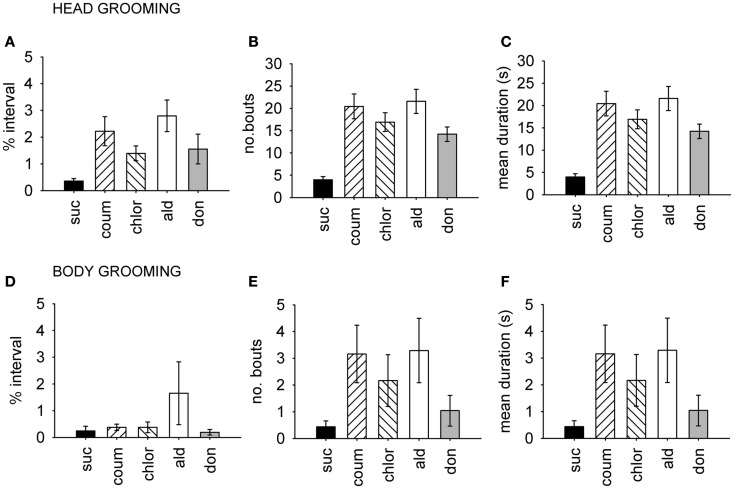
**Time budget, frequency and duration data for grooming behavior**. Head and body grooming are shown separately, and data pooled from both winter and summer. **(A–C)**, Head grooming, **(D–F)** body grooming. **(A,D)** Show time budget data, expressed as the mean percentage of the observation interval (±SEM) spent engaged in each type of grooming. **(B,E)** Show count data, expressed as the mean number of times (±SEM) bees performed the grooming behavior. **(C,F)** Show the mean duration (±SEM) in seconds of each grooming episode. *N* ≥ 23 for each treatment group. Suc, sucrose; Coum, coumaphos; Chlor, chlorpyrifos; Ald, aldicarb; Don, donepezil.

Because the AChE inhibitors had a strong effect on grooming behavior, we examined head and body grooming separately (Figure 3). AChE treatment increased the total time spent grooming the head (MGLM, *F*_4,119_ = 4.10, *P* = 0.004) but not the body (MGLM, *F*_4,119_ = 1.27, *P* = 2.84). Aldicarb (LSD, *P* > 0.001) and coumaphos (LSD, *P* = 0.004) were the compounds which caused significant increases in the percentage of time spent head grooming. The number of head grooming bouts was also affected by AChE treatment (Pois reg., $\chi_{4}^{2}$ = 250, *P* = 0.001). All the drug treatment groups groomed more frequently than the control (*P* > 0.001 for all treatments). The number of body grooming bouts was also greater for AChE inhibitors ($\chi_{4}^{2}$ = 60.9, *P* = 0.001), and again all treatments had an effect (aldicarb, *P* > 0.001; chlorpyrifos, *P* > 0.001; donepezil, *P* = 0.015; coumaphos, *P* > 0.001). The mean duration of each grooming bout was unaffected by AChE inhibitor (MGLM, head grooming, *F*_4,119_ = 0.514, *P* = 0.726; body grooming, *F*_4,119_ = 1.11, *P* = 0.353).

### Coumaphos has dose-dependent and seasonal effects on grooming and abdominal movements

The dose of the AChE inhibitors experienced during prolonged exposure also influenced behavior. We tested the effects of three concentrations of coumaphos on the same behaviors measured in the previous experiment. As before, we used factor analysis to reduce the dimensionality of the data. In this case, the factor analysis produced three factors which accounted for 71.5% of the variation in behavior (Table 2). The factor 1, accounting for 28.3% of the variation, consisted of a positive correlation between both head and body grooming behavior and abdominal spasms. Factor 2 represented an inverse relationship between walking behavior and upside down behavior; and factor 3 represented an inverse relationship between flying behavior and remaining still.

**Table 2:** **Factor analysis produced from whole behavioral dataset**.

	Factor		
	1	2	3
% Variance	28.3%	52.5%	71.5%
Walk	−0.460	−0.827	0.276
Head grooming	0.743	0.137	0.282
Body grooming	0.793	−0.063	−0.037
Still	0.181	0.459	−0.710
Fly	0.122	0.276	0.758
Upside down	−0.055	0.797	0.210
Abdomen spasms	0.735	0.254	−0.223

*Shading indicates which behavioral variables made the largest contribution to each factor. Factors with eigenvalues greater than 1 were extracted from the data. *N* = 88*.

**Figure 4:**
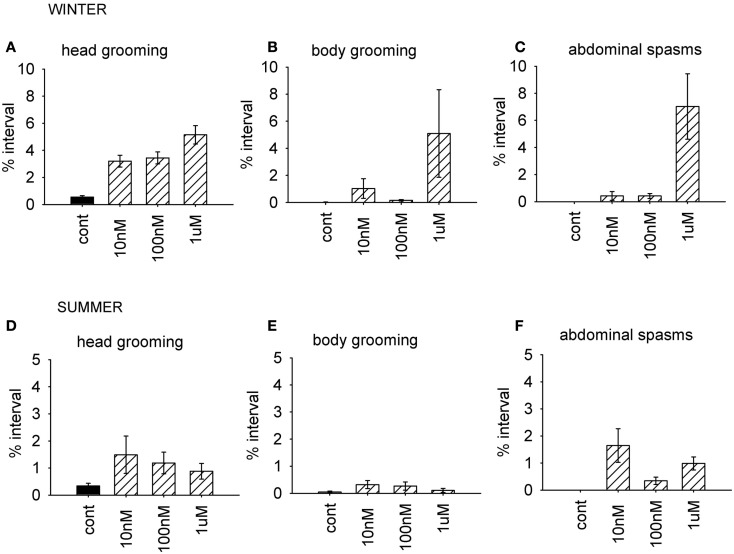
**Time budget data for bees treated with different doses of coumaphos**. Mean percentage of the observation interval (±SEM) spent engaged in the behaviors comprising the behavioral component which was differentially affected by the drugs according to the time of year. **(A)** Head grooming, winter **(B)** body grooming, winter. **(C)** Abdominal spasms, winter. **(D)** Head grooming, summer. **(E)** Body grooming, summer. **(F)** Abdominal spasms, summer. *N* ≥ 10 For each treatment group and time of year. Cont, control; sucrose.

As before, we used the factor scores to examine the influence of the AChE inhibitor on behavior. The time of year of the assay was also included in the analysis. Factor 1 (grooming and abdominal spasms) was affected by both coumaphos concentration and the time of year (MGLM, two-way interaction, *F*_3,87_ = 4.56, *P* < 0.001). The data for these behaviors was therefore separated according to when it was collected, and reanalyzed (Figure 4). In winter, coumaphos treatment affected the expression of these behaviors (GLM, *F*_3,40_ = 8.53, *P* < 0.001), with the 1 μM treatment group performing more of these behaviors than the controls. In summer, however, there was no effect of coumaphos concentration on these behaviors (GLM, *F*_3,46_ = 0.771, *P* = 0.516). Time budget data for the behaviors comprising this factor are shown for both winter and summer bees in Figure 4. The factors representing all the other behaviors were unaffected by coumaphos treatment (factor 2, *F*_3,87_ = 1.219, *P* = 0.308; factor 3, *F*_3,87_ = 0.492, *P* = 0.689).

### AChE inhibition in the brain and gut

**Figure 5:**
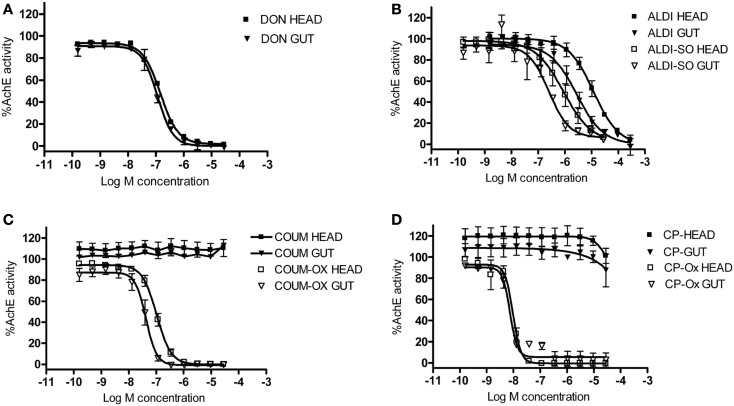
**The effect of AChE inhibitors on honeybee AChE activity**. Inhibition of AChE activity in honeybee head and gut lysates by **(A)** donepezil (DON), **(B)** aldicarb (ALDI) and aldicarb sulfoxide (ALDI-SO), **(C)** coumaphos (COUM), coumaphos oxon (COUM-OX), and **(D)** chlorpyrifos (CP) and chlorpyrifos oxon (CP-OX). AChE activity was normalized to control measurements. Hill equation fits of the data were made using GraphPad Prism. *N* = 3 treatment replicates; three technical replicates were performed for each sample.

Biochemical assays were performed to assess the ability of the AChE inhibitors and their activated metabolites to inhibit AChE extracted from honeybee brain and gut tissue. Graphs showing percentage AChE activity after incubation with a range of inhibitor concentrations are shown in Figure 5. Chlorpyrifos oxon and coumaphos oxon, and aldicarb sulfoxide (the active metabolites of each AChE inhibitor) showed far greater AChE inhibition than the parent compounds; this result was expected, because organophosphates and carbamates are metabolically activated *in vivo* to produce these more potent compounds (Dauterman, 1971; Fukuto, 1990). IC_50_ values for the active compounds are shown in Table 3.

**Table 3:** **IC_50_ values for AChE inhibitors**.

	Brain	Gut
Coumaphos oxon	109.10 ±17.10	43.05 ±7.87
Chlorpyrifos oxon	5.23 ± 2.30	5.11 ± 0.33
Aldicarb sulfoxide	1101.48 ± 166.51	439.45 ± 66.01
Donepezil	151.19 ± 4.47	115.03 ± 5.86

*IC_50_ values were calculated using GraphPad Prism from the dose-response curves shown in Figure 5. Mean IC_50_ values are expressed in nanomolar concentrations; SEMs are shown in brackets. *N* = 3 biological replicates for each compound; three technical replicates were performed for each sample*.

Chlorpyrifos oxon was clearly the most potent inhibitor of AChE, and was equally potent on both tissues. For the other AChE inhibitors, there was a difference in IC_50_ depending on which tissue was assayed; the IC_50_ values were lower for the gut tissue than for the brain, meaning that the AChE inhibition effect was greater in the gut tissue (two-tailed *t*-test; coumaphos oxon and aldicarb sulfoxide, *P* > 0.01; donepezil, *P* > 0.001).

### AChE gene transcription increases in the brain and the gut in response to AChE inhibitors

**Figure 6:**
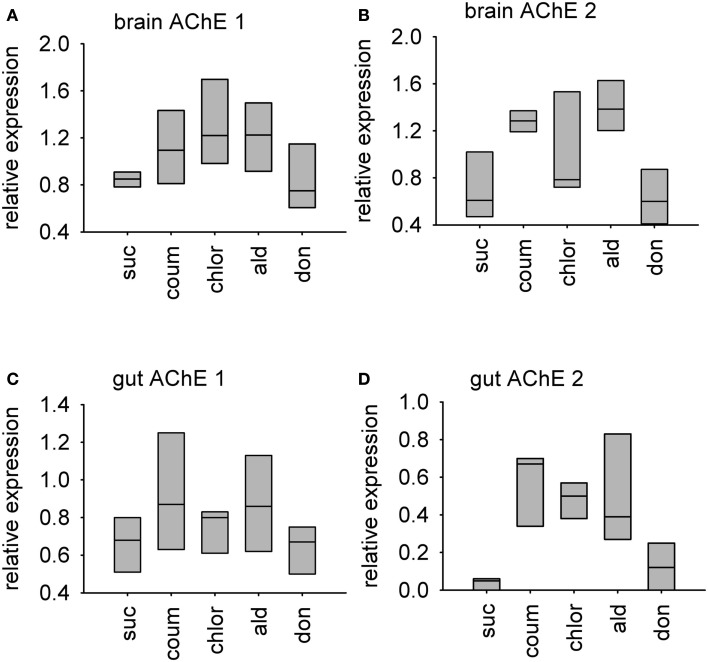
**Relative levels of two AChE encoding transcripts in AChE inhibitor treated bees**. A box-and-whisker plot of the median levels of AChE transcripts is shown. Levels are expressed relative to expression of beta-tubulin control transcript in the same tissue sample. *N* = 3 treatment replicates, with guts from three individuals or brains from five individuals collected from eat treatment group. **(A)** AChE-1 transcript in brain tissue **(B)** AChE-2 transcript in brain tissue **(C)** AChE-1 transcript in gut tissue **(D)** AChE-2 transcript in gut tissue. Suc, sucrose; Coum, coumaphos; Chlor, chlorpyrifos; Ald, aldicarb; Don, donepezil.

Relative transcript abundance of AChE-1 (accession number XM_393751) and AChE-2 (accession number NM_001040230) was measured in the brain and gut tissue from bees used in the behavioral experiments. Graphs showing median relative expression data are shown in Figure 6. Transcript levels of AChE-1 in the brain, but not the gut, were affected by AChE inhibitor treatment (median test, brain, $\chi_{4}^{2}$ = 10.0, *P* = 0.040; gut, $\chi_{4}^{2}$ = 4.29, *P* = 0.369). Chlorpyrifos was the only AChE inhibitor to affect AChE-1 expression in the brain (*post hoc*, *P* = 0.05). Exposure to AChE inhibitors had a stronger influence on expression levels of AChE-2 transcripts in both brain and gut tissue (median test, brain, $\chi_{4}^{2}$ = 14.0, *P* = 0.007; gut, $\chi_{4}^{2}$ = 9.64, *P* = 0.047). In brain tissue, AChE-2 transcript levels increased in response to coumaphos and aldicarb (*post hoc*, *P* = 0.05), but levels in donepezil and chlorpyrifos treated bees were not significantly different to the controls (*post hoc*, *P* = 1.00, *P* = 0.157). (It should be noted that AChE-2 levels were much more variable in the chlorpyrifos treated bees than in the other treatment groups). In gut tissue, AChE-2 levels increased in response to treatment with coumaphos (*post hoc, P* = 0.015), chlorpyrifos (*post hoc*, *P* = 0.032), and aldicarb (*post hoc*, *P* = 0.032), but not by donepezil (*post hoc*, *P* = 0.715).

## Discussion

Exposure to sub-lethal doses of AChE inhibiting pesticides altered honeybee behavior, increasing the amount and frequency of grooming behavior, and impairing the ability to perform the righting reflex. Abdominal spasms were also seen, particularly in bees fed high doses of coumaphos. Exposure to AChE inhibitors increased expression of the AChE-2 gene in both the brain and the gut. Whether the bees were sampled from the outdoor summer colony, or the indoor winter colony, also had an effect on the expression of the behaviors associated with the AChE inhibitors.

As observed in our experiments, exposure to AChE inhibitors causes acute disruptions of motor function in other animals, including rats, fish, and flies (Miller and Kennedy, 1972; Moser, 1995; Patil and David, 2010). Organophosphate and carbamate exposure causes tremors and unusual gait in rats, and uncoordinated movement and bouts of paralysis in flies (Miller and Kennedy, 1972; Moser, 1995). Fish treated with organophosphates are reported to exhibit bouts of hyperactive, erratic movement, along with a tendency to sink to the bottom of their tank (Patil and David, 2010). As observed in these animals, we also found that motor function was altered by AChE inhibitors: bees walked less, were more likely to have difficulty righting after falling over, and exhibited strange abdominal movement and spasms. Although we did not measure it, the effect of AChE inhibitors on motor function could also be reflected in a disruption in flight and foraging behavior. In fact, this effect could be especially profound for foragers, as they exhibit lower levels of AChE in the brain (Shapira et al., 2001) and so could be more likely to be affected by long-term AChE inhibition. Acute exposure to AChE inhibitors improves learning and memory by enhancing cholinergic transmission in honeybees (Shapira et al., 2001; Guez et al., 2010; Williamson et al., 2012). The effects of chronic exposure to AChE inhibitors on higher cognitive function in bees have not yet been studied but will be important for assessing the impact of these substances on foraging and navigation.

Bees exposed to AChE inhibitors also spent more time grooming. This is especially important because of the widespread and deliberate application of coumaphos in honeybee colonies to combat *Varroa* mites. The increase in grooming behavior caused by AChE inhibitors could improve the efficacy of coumaphos as a mite treatment (Milani and Iob, 1998) but at a cost, as exposure also produces abdominal spasms. The abdominal spasms were often also accompanied by both the expulsion of fecal material, and regurgitation of crop contents, suggesting that gut function was significantly disrupted. The effect of AChE inhibitors, especially coumaphos, on the honeybee gut may explain why bees reared in combs containing high levels of acaricides often also have heavy infestations with the gut parasite *Nosema ceranae* (Wu et al., 2012). It has previously been reported that food assimilation and defecation in the freshwater prawn *Macrobrachium malcomsonii* is also disrupted by a carbamate pesticide (carbaryl), which may suggest a common cholinergic mechanism regulating arthropod gut functions (Bhavan et al., 2011).

The AChE activity assays demonstrated that metabolites of coumaphos, chlorpyrifos, and aldicarb all inhibit AChE in both brain and gut tissue from honeybees. Although it is of course difficult to correlate environmental levels of the parent compound with levels of the active metabolite in the tissues of live bees, it is perhaps reasonable to predict that recorded levels of organophosphates and carbamates in comb wax and stored pollen could cause AChE inhibition in bees inhabiting a contaminated hive (Mullin et al., 2010; Wu et al., 2011). The AChE inhibiting effects of the organophosphates are of particular concern, given the high levels of coumaphos exposure which can accumulate where it is used as a mite treatment (Mullin et al., 2010), and given the potent AChE inhibiting activity of chlorpyrifos oxon at even very low concentrations. An interesting finding was that the AChE inhibiting activity of donepezil and of the coumaphos and aldicarb metabolites was more potent on extracts from gut tissue than brain tissue. Direct inhibition of AChE in the gut provides an explanation for the abdominal spasms and disrupted gut function we observed in the behavioral experiments. The differences between AChE inhibitor potency in the gut and brain tissue extracts are more difficult to explain, and it is unclear what physiological relevance this finding may have. It may suggest that levels of the different AChE enzymes differ between tissues; previous studies have identifies at least two different AChE proteins in the honeybee (Belzunces et al., 1988; Badiou et al., 2007).

Unfortunately, we were unable to measure transcript levels by RT-PCR, and so cannot be absolutely certain of the level of change expression, but our results strongly suggest that long-term exposure to AChE inhibitors results in upregulation of transcription of both AChEs in bees. This change did not depend on whether the AChE inhibitor was an organophosphate or a carbamate compound. Interestingly, donepezil consistently affected behavior, and AChE gene transcription, to a lesser extent than the other compounds, despite the IC_50_ values from the AChE activity assay described above indicating that it readily inhibits bee AChE activity. This could be explained by the fact that the inhibition of AChE by donepezil is readily reversible, over a time scale of minutes; whereas, the effects of aldicarb are reported to be reversible only over several hours (Baron, 1994; Seltzer, 2005). Furthermore, coumaphos and chlorpyrifos, irreversibly bind AChE, requiring the synthesis of new enzyme before the effects are ameliorated (Fukuto, 1990; Pohanka, 2011). In addition, the AChE inhibitors had different ways of binding to the enzyme: organophosphate and carbamate pesticides act at the active site of the enzyme, but donepezil binds at a peripheral anion site (Fukuto, 1990; Pohanka, 2011).

Animals may adapt to pesticide exposure by short-term physiological adaptations for detoxification and compensation, and by longer-term population-level adaptations such as genetic changes which confer reduced sensitivity (Ramphul et al., 2009; Bass and Field, 2011; Boncristiani et al., 2012). AChE transcript levels have been observed to change in vertebrate species after exposure to organophosphate compounds: in the fish *Panasianodon hypopthalmus*, AChE transcript levels in the liver decreased after trichlorfon exposure, whereas in rats, AChE transcript levels in the brain increased after exposure to sarin (Damodaran et al., 2003; Sinha et al., 2010). Our experiments show that AChE transcript levels also change in the honeybee in response to organophosphate and carbamate exposure, suggesting that this may be used as a potential biomarker for exposure to these classes of pesticide.

Over longer timescales, increased AChE levels as a heritable trait rather than a transient response are a common mechanism of resistance to organophosphate pesticides (Kwon et al., 2010; Shang et al., 2012). Organophosphate resistant spider mites *Tetranychus urticae* have several duplications of an AChE encoding gene (Kwon et al., 2010). This also occurs in insects, where duplication of the AChE-2 gene is associated with organophosphate resistance in the aphid *Aphis gossypii* (Shang et al., 2012). Another resistant strain of the same aphid species also shows decreased expression of AChE-1, and increased expression of AChE-2 (Pan et al., 2010). This is interesting in the context of AChE-1 being the main AChE transcript in the nervous system, and the known target of organophosphate pesticides, in the beetle *Tribolium castaneum*; whereas the function of AChE-2 is less clear (Lu et al., 2012). In the context of these previous studies, and the data we present here, suggests that the increased expression of AChE-2 may be an adaptive mechanism by which insects counteract the inhibition of AChE-1 by organophosphate and carbamate pesticides.

## Conflict of Interest Statement

The authors declare that the research was conducted in the absence of any commercial or financial relationships that could be construed as a potential conflict of interest.
